# The Challenge of Choosing the Best Classification Method in Radiomic Analyses: Recommendations and Applications to Lung Cancer CT Images

**DOI:** 10.3390/cancers13123088

**Published:** 2021-06-21

**Authors:** Federica Corso, Giulia Tini, Giuliana Lo Presti, Noemi Garau, Simone Pietro De Angelis, Federica Bellerba, Lisa Rinaldi, Francesca Botta, Stefania Rizzo, Daniela Origgi, Chiara Paganelli, Marta Cremonesi, Cristiano Rampinelli, Massimo Bellomi, Luca Mazzarella, Pier Giuseppe Pelicci, Sara Gandini, Sara Raimondi

**Affiliations:** 1 Department of Experimental Oncology, IEO European Institute of Oncology IRCCS, via Adamello 16, 20139 Milan, Italy; federica.corso@ieo.it (F.C.); giulia.tini@ieo.it (G.T.); luca.mazzarella@ieo.it (L.M.); piergiuseppe.pelicci@ieo.it (P.G.P.); 2 Department of Mathematics (DMAT), Politecnico di Milano, via Edoardo Bonardi 9, 20133 Milan, Italy; 3 Centre for Analysis, Decision and Society (CADS), Human Technopole, via Cristina Belgioioso 171, 20157 Milan, Italy; 4 Medical Physics Unit, IEO European Institute of Oncology IRCCS, via Ripamonti 435, 20141 Milan, Italy; giuliana.lopresti@ieo.it (G.L.P.); francesca.botta@ieo.it (F.B.); daniela.origgi@ieo.it (D.O.); 5 Department of Electronics, Information and Bioengineering (DEIB), Politecnico di Milano, via Ponzio 34, 20133 Milan, Italy; noemi.garau@ieo.it (N.G.); chiara.paganelli@polimi.it (C.P.); 6 Division of Radiology, IEO European Institute of Oncology IRCCS, via Ripamonti 435, 20141 Milan, Italy; cristiano.rampinelli@ieo.it (C.R.); massimo.bellomi@ieo.it (M.B.); 7 Molecular and Pharmaco-Epidemiology Unit, Department of Experimental Oncology, IEO European Institute of Oncology IRCCS, via Adamello 16, 20139 Milan, Italy; simonepietrodeangelis@gmail.com (S.P.D.A.); federica.bellerba@ieo.it (F.B.); sara.gandini@ieo.it (S.G.); 8 Radiation Research Unit, IEO European Institute of Oncology IRCCS, via Giuseppe Ripamonti 435, 20141 Milan, Italy; lisa.rinaldi@ieo.it (L.R.); marta.cremonesi@ieo.it (M.C.); 9 Department of Physics, University of Pavia, via Bassi 6, 27100 Pavia, Italy; 10 Clinica di Radiologia EOC, Istituto Imaging della Svizzera Italiana (IIMSI), via Tesserete 46, 6900 Lugano, Switzerland; stefania.rizzo@eoc.ch; 11 Division of Early Drug Development for Innovative Therapies, IEO European Institute of Experimental Oncology IRCCS, via Ripamonti 435, 20141 Milan, Italy; 12 Department of Oncology and Hematology-Oncology, University of Milan, via Festa del Perdono 7, 20122 Milan, Italy

**Keywords:** radiomics, machine learning, classification, simulation, sample size, balancing, signal, feature selection, CT images, lung cancer

## Abstract

**Simple Summary:**

Radiomics aims to extract high-dimensional features from clinical images and associate them to clinical outcomes. These associations may be further investigated with machine learning models; however, guidelines on the most suitable method to support clinical decisions are still missing. To improve the reliability and the accuracy of radiomic features in the prediction of a binary variable in a lung cancer setting, we compared several machine learning classifiers and feature selection methods on simulated data. These account for important characteristics that may vary in real clinical datasets: sample size, outcome balancing and association strength between radiomic features and outcome variables. We were able to suggest the most suitable classifiers for each studied case and to evaluate the impact of method choices. Our work highlights the importance of these choices in radiomic analyses and provides guidelines on how to select the best models for the data at hand.

**Abstract:**

Radiomics uses high-dimensional sets of imaging features to predict biological characteristics of tumors and clinical outcomes. The choice of the algorithm used to analyze radiomic features and perform predictions has a high impact on the results, thus the identification of adequate machine learning methods for radiomic applications is crucial. In this study we aim to identify suitable approaches of analysis for radiomic-based binary predictions, according to sample size, outcome balancing and the features–outcome association strength. Simulated data were obtained reproducing the correlation structure among 168 radiomic features extracted from Computed Tomography images of 270 Non-Small-Cell Lung Cancer (NSCLC) patients and the associated to lymph node status. Performances of six classifiers combined with six feature selection (FS) methods were assessed on the simulated data using AUC (Area Under the Receiver Operating Characteristics Curves), sensitivity, and specificity. For all the FS methods and regardless of the association strength, the tree-based classifiers Random Forest and Extreme Gradient Boosting obtained good performances (AUC ≥ 0.73), showing the best trade-off between sensitivity and specificity. On small samples, performances were generally lower than in large–medium samples and with larger variations. FS methods generally did not improve performances. Thus, in radiomic studies, we suggest evaluating the choice of FS and classifiers, considering specific sample size, balancing, and association strength.

## 1. Introduction

Radiomics focuses on extracting and mining high-dimensional sets of quantitative features from medical images, which are expected to provide a detailed and comprehensive characterization of the tumor phenotype [1], being calculated on the entire volume of the lesion. Indeed, radiomic features are also hypothesized to supply crucial information regarding tumor physiology [2]. Radiomics uses either a set of predefined engineered features [3] that describe the lesion shape, its gray-level intensity, and its texture or, alternatively, features that can be automatically “deep learned” from images [4]. Promising associations of radiomics with the underlying biology of specific tumors and/or the clinical outcomes, including histopathology, disease-free survival, overall-free survival, response to therapies, and diagnosis [2,5,6,7,8,9,10] have led to a rapid expansion in this field, opening new avenues to investigate the clinical utility of radiological medical imaging [11]. As proof of the exceptionally increasing interest, a simple search in PubMed using “radiomics” as the keyword identified in 2020 a number of papers around 40 times higher than in 2015.

Predictive and prognostic models characterized by high accuracy, reliability, and efficiency are vital factors for radiomics to play an active role in supporting clinical decisions in oncology [12,13,14,15,16,17]. Several statistical models have been researched and applied so far, from simple linear regression and curve-fitting to advanced machine learning (ML) methods, such as decision trees, support vector machines, random forests, boosted trees, or neural networks [6,16,18,19,20,21]. Most of the published studies have assessed the predictive capabilities of radiomic features [22,23,24,25]. However, fewer papers have focused on the impact of different methods, such as feature selection and classification, on predictive modelling. In truth, different approaches may highly influence the variability of prediction scores, thus affecting the reproducibility of the results in other studies. The choice of the classification method was suggested to be the major source of performance variation [6,8,26,27]. Thus, the identification of the optimal ML methods for radiomic applications represent a crucial step toward stable and clinically relevant radiomic biomarkers.

For lung cancer, few publications compared the performance of different methods for the prediction of binary outcomes by using patient data [6,8,28]. However, these studies have a limited sample size and the real underlying feature–outcome association structure is actually unknown, thus restricting the interpretation and the selection of the best-performing model, which may also be related to the frequency of the investigated outcome, its strength of association and to the number of radiomic features included. These issues can make it difficult to generalize the results for other clinical contexts. Especially as the association with the outcome in a dataset is something not known a priori, since it measures how much (high/low) data variability the radiomic features can explain and thus predict the outcome expression. External validation represents a decisive test of the model’s robustness, but has only been performed in a few cases [6]. A widely used strategy to account for all the aspects mentioned before, but never applied in radiomics, is the generation of synthetic datasets using a customized simulation procedure, to achieve adequate statistical power for detecting performance differences among models.

Starting from the first radiomic study by Aerts et al. [2], numerous further studies were conducted to evaluate radiomic features of Computed Tomography (CT) images in a lung cancer setting, therefore the combination of this imaging technique and cancer type is one of the most investigated in the literature, with the most data collected so far. In our institute, we previously obtained data on around 300 patients with Non-Small-Cell Lung Cancer (NSCLC) to investigate the association between radiomic features of CT images and lung cancer outcomes (lymph node involvement and survival) [29].

In the present study, we therefore explored the characterization of optimal classification approaches applied to CT images of NSCLC patients, implementing a classification framework on both real and synthetic data, trying to account for any possible drawbacks related to both the underlying structure of the real dataset and the simulation algorithm itself. The clinical outcome was the lymph node involvement, which can be treated as a dichotomous variable (presence/absence), allowing our results to be easily extended to any binary clinical context.

## 2. Materials and Methods

### 2.1. Dataset

#### Non-Small-Cell Lung Cancer (NSCLC) Patients’ Original Dataset

A retrospective study recently published by our group [29] collected and analyzed preintervention CT images of 270 early-stage NSCLC patients (up to pT3N1) operated on in our Institute between 2012 and 2016. The Institutional Review Board approved the study (UID 2172) waiving the need for informed consent. Information about the presence/absence of positive lymph nodes, pathologically assessed after surgery, was available for all patients: 71 (26%) of them showed positive lymph nodes (pN = 1), while the remaining 199 (74%) had a negative lymph node status (pN = 0). Further information about the original dataset is available in Table S1. CT images were acquired on four different scanners after contrast medium injection (portal phase). Image reconstruction was performed with two different algorithms: Filtered Back Projection (FBP) for a total of 187 patients (69%) and Iterative Reconstruction (IR) for the remaining 83 patients (31%). A total of 881 radiomics features were extracted from CT images with the IBEX v 1.0 β tool. After reproducibility and stability analysis, a subset of 168 radiomic features was selected to be included in the main analyses. More details about image acquisition, radiomic features extraction and selection can be found in [29].

### 2.2. Simulated Datasets

#### 2.2.1. Radiomic Features Simulation

**Figure 1:**
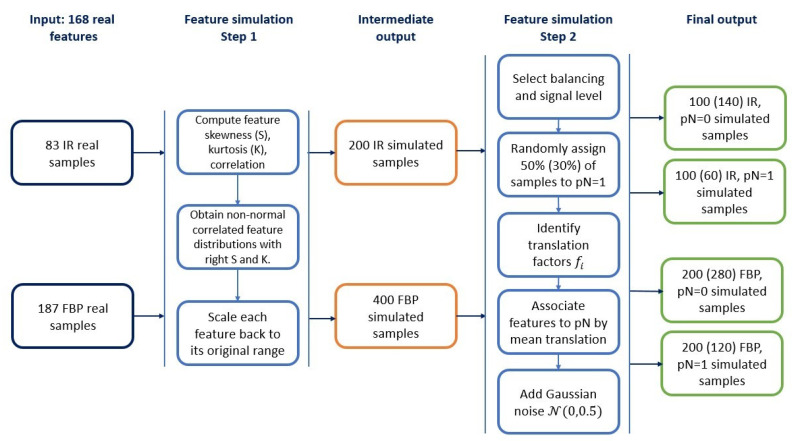
Simulation procedure flow chart. Input are two sets of 168 real features, derived from CT (Computer Tomography) images reconstructed with the Filtered Back Projection (FBP) and Iterative Reconstructions (IR) algorithm, respectively (left column). The first step of the procedure (second column) describes how to obtain simulated features not associated to the outcome, which represents an intermediate output (middle column). The second part of the procedure (fourth column) associates samples and features to the outcome variable and provides as the final output, different sets of 168 features (right column). Cardinality of those sets depends on algorithm and sample balancing: sizes for balanced cases are reported, with sizes for unbalanced cases in parenthesis.

Starting from the NSCLC data described above, we simulated radiomic features to build synthetic controlled scenarios. Details on radiomic data simulation are reported in the Supplementary Materials. Briefly, our simulation procedure was divided into two main steps (Figure 1): (1) Simulation of 168 features without association to the outcome; (2) Selection of balancing and signal level and the association of the simulated features and samples to the outcome.

We applied both procedures separately to FBP and IR-derived features, since the algorithm was found to be one of the major issues influencing the lesion texture in the image [29]. In the first step, we simulated 168 multivariate non-normal distributions starting from the correlation matrix, skewness and kurtosis of the real NSCLC features as suggested by Vale and Maurelli [30]. Since the ranges of the radiomic features may vary a lot, we translated the obtained variables to their original ranges.

In the second main step of the simulation procedure, we associated samples and features to different outcome classes and we introduced the possibility to create balanced/unbalanced datasets with features carrying a high or low association signal to the outcome. First, we chose the sample balancing according to an equal (balanced) or unequal (unbalanced) distribution of the outcome classes. In the former case we randomly assigned 50% of the samples to the class pN = 1, in the latter only 30%. Two preliminary sets of data, each one consisting of 600 simulated patients’ data, were therefore created. We then associated the features to the outcome to generate the signal (high or low, according to the strength of association with the outcome) able to separate samples with a different outcome class. We chose three features that were found to be significantly associated with the positive lymph node in the original dataset [29]: ClusterShade from GLCM25 category calculated along a 135° direction with four voxels offset (F1), the 70th percentile of the intensity values in the cumulative histogram (F2), and the maximum diameter evaluated on the 3D lesion volume (F3).

Four datasets including 600 simulated patients each were eventually obtained, according to the combination of balancing (balanced/unbalanced) and signal (high/low) (Figure 1).

#### 2.2.2. Construction of Simulated Datasets

We first split the four main datasets into a training set (2/3 of the samples, *N* = 400 patients) and validation set (1/3 of the samples, *N* = 200 patients). Keeping the division between the training and validation set, we built eight additional datasets with lower dimensionality (four with *N* = 300 and four with *N* = 100) by simple random sampling, taking into account the original proportions between FBP and IR groups (Table 1). The balanced (50–50%) and unbalanced (70–30%) proportions between pN = 0 and pN = 1 were guaranteed by subsampling the two classes separately both in the training and validation sets.

### 2.3. Feature Selection and Classifier Methods

Six different ML classifiers, combined with five feature selection (FS) methods or no feature selection step, were investigated (Table 2) and applied to the twelve simulated scenarios, with the final aim to correctly classify samples associated to a binary outcome variable. Details on FS and classifier methods are reported in the Supplementary Materials.

### 2.4. Classification of Simulated and Real Datasets

#### 2.4.1. Classification Framework

We designed a systematic classification framework which consists of canonical steps including: preprocessing, cross-validation, performance evaluation and classification. Finally, we characterized the model with the optimal prediction metrics on the validation set and we summarized the performance of each classifier and FS method across the different scenarios. The main steps of the classification framework are visualized in Figure S1 and detailed in the Supplementary Materials. All the models were implemented using the package “caret” [31] of software R (version 4.0.3).

#### 2.4.2. Application to Simulated and NSCLC Data

We applied the proposed classification framework to the twelve simulated scenarios as well as to the original NSCLC data. In order to summarize the overall performances of FS methods and classifiers, we calculated the mean values and standard deviations of the Area Under the Receiver Operating Characteristics Curves (AUC), sensitivity (SE) and specificity (SP) among all scenarios for each classifier. In a second step, we also conditioned these measures to the signal strength. We used the same approach to study the stability of the FS methods. We also performed a Wilcoxon two-independent sample (α = 0.05) test to compare the average AUC with and without the FS step.

For four out of the six different classifiers it was possible to retrieve a list of the top twenty features, obtained according to a selection criterion or measure of importance. According to this ranking, we observed if the three “control” features, associated with the clinical outcome by design, were correctly selected by each combination of the FS and ML models.

To evaluate the results of our simulation study on real data, we also applied our pipeline to the original data of 270 patients affected by NSCLC. To obtain an application of our models, independent from the previously published results [29], we performed a new random split of the dataset in the training (*n* = 175) and validation (*n* = 95) sets. Since the real dataset was characterized by an unbalanced proportion of positive lymph nodes (30%), a SMOTE (Synthetic Minority Over-sampling) [32] oversampling technique was applied. We applied the pipeline to evaluate the best classifier and FS method in the prediction of a binary variable represented by the classes pN = 0 and pN = 1.

## 3. Results

### 3.1. Data Simulation Step

To identify the main issues that should be tackled when simulating radiomic features, we first carried out some descriptive analyses of our real data on NSCLC patients.

We separately explored radiomic features obtained from CT images reconstructed with FBP (168 features) and IR (168 features) algorithms. We observed that only 107 features of the total 336 features derived from both algorithms (32%), were normally distributed. Additionally, as expected, the correlation among features was high and we were able to keep a similar correlation structure in the simulated data, although with a lower overall correlation (Figure S2).

The association with the lymph node status, carried out as the second step of the simulation procedure, was computed by modelling the association for the three selected features F1, F2 and F3 as low or high. The feature distributions in the positive and negative classes were separated by specific factors, large enough to significantly associate all (high signal) or some (low signal) of them to the positive lymph node status (Table S2).

### 3.2. Classification Performances on Balanced Samples

**Figure 2:**
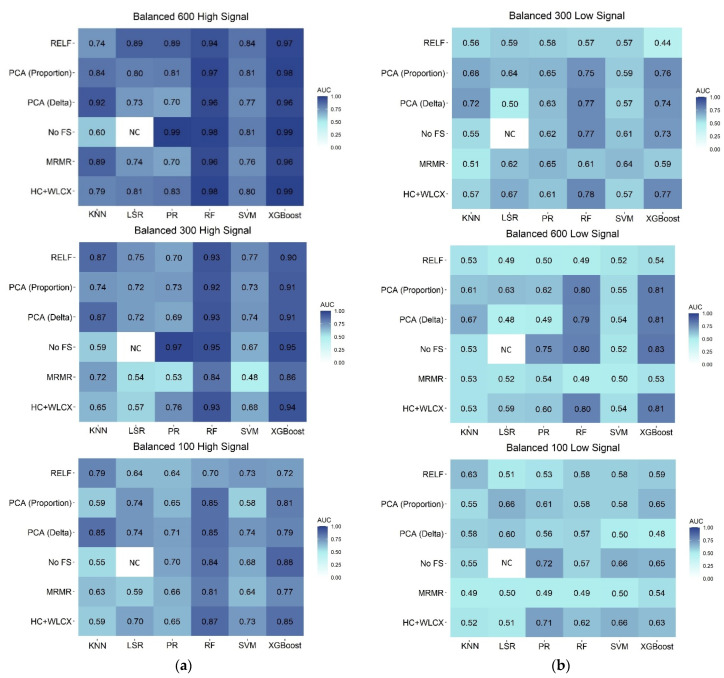
Heatmap representing the predictive performance (AUC) of feature selection (rows) and classification (columns) methods in the validation set for: (**a**) simulated samples with a high signal; (**b**) simulated samples with a low signal. HC + WLCX = Hierarchical clustering + Wilcoxon; KNN = K-Nearest Neighbor; LSR = Logistic Step-wise Regression; MRMR = Minimum Redundancy Maximum Relevance; PCA (Delta) = Principal Component Analysis clustering with Delta plot stop criterion + Wilcoxon; PCA (Proportion) = Principal Component Analysis clustering with proportion stop criterion + Wilcoxon; PR = Penalized Regression; RF = Random Forest; RELF = Relief; XGBoost = Extreme Gradient Boosting; SVM = Support Vector Machine.

The predictive performance of the six ML algorithms combined with the six FS methods in the validation set is reported in Figure 2 for the balanced scenarios.

When the association signal was high, almost all the classification algorithms and FS methods presented good performances (Figure 2, panel A), generally with a slight decrease in the AUC values when the sample size was diminished. Nevertheless, RF (Random Forest) and XGBoost (Extreme Gradient Boosting) maintained excellent performances for all the sample sizes. AUC values for those methods, across FS, ranged from 0.94 to 0.99 for the large sample, from 0.84 to 0.95 for the medium, and from 0.70 to 0.87 for the small sample, respectively. When no FS step was performed, PR (Penalized Regression) also obtained a comparable high performance in the large and medium datasets (AUC = 0.99 and 0.97, respectively).

Noticeable differences in classification performances arose instead for the balanced samples with a low association to the outcome (Figure 2, panel B). In particular, for large and medium samples, RF and XGBoost were still the methods with the best performances when combined with FS methods different from RELF (Relief) and MRMR (Minimum Redundancy Maximum Relevance): the AUC ranged from 0.79 to 0.83 for both large and medium samples. For the small sample, the best performances were observed for PR with no FS step (AUC = 0.72) or HC + WLCX (Hierarchical clustering + Wilcoxon) (AUC = 0.71). In all the other cases, classifiers returned similarly poor performances, regardless of the FS method, with the AUC generally between 0.40 and 0.70 for all the sample sizes.

The ability of each approach to correctly select the features with a real, but low, association with the outcome is represented in Supplementary Figure S3, Panel A. For the large and medium sample, RF and XGBoost, combined with FS methods different from RELF and MRMR, were able to select the three features associated to the outcome among the top 20 more relevant features. For the small sample, all the considered classification methods were able to identify two out of the three associated features when combined with PCA (Delta) + WLCX (Principal Component Analysis clustering with Delta plot stop criterion + Wilcoxon). With this FS method, PR was able to identify all three of the associated features as the most important in both large and medium samples. Looking at FS methods, RELF was the worst in recovering important features in the large and medium samples, while high variability was observed in the small sample.

### 3.3. Classification Performances on Unbalanced Samples

Since the main concern for unbalanced samples is a good estimate of the less represented class, for the unbalanced samples we measured performances of classifiers and FS methods with sensitivity (SE) and specificity (SP), in addition to AUC.

**Figure 3:**
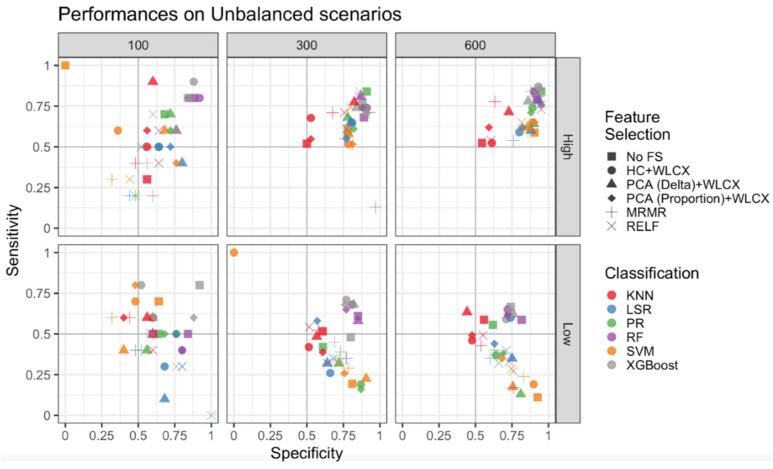
Sensitivity and Specificity for unbalanced datasets. Performances of the classification and feature selection methods applied to the unbalanced cases are displayed for a high signal (upper panels) and a low signal (lower panels). Columns report results for the different sample sizes. Colors are used to distinguish classification algorithms, shapes for feature selection methods.

We first compared results obtained for unbalanced samples with and without SMOTE correction and observed that SE was improved overall after the correction [32] (Figures S4–S6). Thus, we applied the SMOTE technique to all the unbalanced scenarios and reported SE and SP obtained according to different sample sizes and the strength of association for unbalanced samples (Figure 3).

When the association signal was high, all the classification and FS methods clustered in the top right panel (SE and SP ≥ 0.50) for medium and large samples, with small differences among FS methods and the highest SE and SP for the classification methods RF, XGBoost and PR. The lowest performance was observed for the KNN (K-Nearest Neighbor) method, with the highest SE and SP (about 0.75) obtained with the PCA (Delta) + WLCX method. High SP and lower SE were obtained by SVM (Support Vector Machine) and LSR (Logistic Step-Wise Regression), suggesting that they are not able to well classify the less represented classes. Finally, for the small sample size there was a high variability of SE and SP across FS and classification methods, with the best performances observed for RF and XGBoost.

Regarding the low signal scenarios, higher SP (≥0.5) was generally achieved for the medium and large scenarios, while the maximum SE was markedly lower than in the high signal scenarios (≤0.75). XGBoost and RF performances were confirmed as the optimal predictive models for large and medium samples, while SVM and PR maintained an SP as good as for the high signal, but SE drastically dropped below 0.5, while KNN had a medium performance. XGBoost without feature selection provided the best trade-off between SE and SP for the small dataset, although the total results showed a wide variability.

In terms of AUC, predictive performances of classifiers and FS methods in the validation set are reported in Figure S7. The overall results are generally comparable to those previously described for balanced samples.

The ability of each approach to correctly select the features with a real, but low, association with the outcome is represented in Figure S3, Panel B. With feature selection methods different from RELF and MRMR, RF and XGBoost (the latter only in combination with a preselection method) were able to select in the group of the top 20 more relevant features, the three features that were indeed simulated as associated with the outcome for the large samples. For the medium and small samples, all the considered classification methods, except LSR, were able to identify two out of the three associated features when combined with PCA (Delta) + WLCX. With this FS method, PR applied to the large sample identified the three features associated to the outcome as the most important ones. Looking at FS methods, RELF was the worst in recovering important features in the large and small samples, while high variability was observed in the medium sample.

### 3.4. Overall Comparison of Feature Selection and Classification Methods

The overall performances of classification algorithms and FS methods are represented in Table 3. Independently from the signal strength, the classifiers giving the best results in terms of mean AUC, SE and SP are RF and XGBoost, while the lowest performance can be attributed to SVM/KNN according to mean values of AUC (0.63/0.63), SE (0.44/0.43) and SP (0.68/0.65) (Table 3).

Performances of different FS methods are more similar across different scenarios. Overall, independently from the signal strength, the method giving slightly better results in terms of mean AUC, SE and SP was the no FS method, while the lowest performance can be attributed to MRMR/RELF according to mean values of AUC (0.63/0.67), SE (0.41/0.44) and SP (0.14/0.13) (Table 4). Overall, no FS reported a borderline better AUC than all the other FS methods combined (AUC = 0.72 vs. 0.68, *p*-value = 0.08). This difference becomes significant when only classification methods including a selection step are considered (PR, RF and XGBoost; AUC = 0.81 vs. 0.73 for no FS and all the other FS methods combined, *p*-value = 0.0007). Interestingly, opposite results were observed for the SVM and KNN methods, that do not include an FS step (AUC were, respectively, 0.58 and 0.65 for no FS compared to any FS, *p*-value = 0.007). These latter results were less strong, but the trend was confirmed, even when the two worst FS methods (MRMR and RELF) were excluded.

### 3.5. Application to NSCLC Real Data

To validate the relevance of our optimization strategy based on simulated data, we applied the methods on our previously published real dataset [29] (described in Supplementary Table S1). The results of model performances are reported in Table 4. Overall, the best performances of tree-based methods (XGBoost and RF) were confirmed by AUCs between 0.67 and 0.69 coupled with the following feature selection methods: HC + WLCX (for RF), HC + PCA (Proportion) and HC + PCA (Delta) (for XGBoost). Good performances were also provided by XGBoost (AUC = 0.67) and LSR (AUC = 0.69) with RELF as the feature selection method. The AUC was slightly lower when no FS was applied. In accordance with previous results about medium unbalanced datasets, SP was higher than SE up to a maximum of 0.94 in PR + RELF, whereas, with some exceptions in RELF, SE generally ranged from 0.33 to 0.67.

## 4. Discussion

In our work we constructed an adequate synthetic dataset, starting from real radiomics data, to study binary classification performances of six different ML algorithms in combination with several FS methods. Using this strategy, we showed that the tree-based methods RF and XGBoost generally performed better than other classifiers, regardless of sample size, strength of the radiomic features–outcome association and sample balancing. The lowest performances were instead observed for the SVM and KNN methods. The performances of the tree-based classifiers for medium and large samples (300 and 600 patients) were very high when the association signal was high (AUC ≥ 0.84) and remained good when the association signal was low (AUC ≥ 0.73 for almost all the FS methods). With a high signal, PR also, with no FS step, obtained a comparable AUC in the large and medium datasets. It should be highlighted that PR has the advantages of a small computational time and higher interpretability: the latter is especially relevant in clinical application, where an interpretable model could be replicated on external validation datasets. On the contrary, RF, and especially XGBoost, have higher computational times and a less immediate interpretation of the results, since they give less emphasis to the biological and clinical significance of the possible association between specific image characteristics and the outcome. Therefore, in an optimal scenario (i.e., high association signal and hundreds of patients) PR provides the best trade-off between performance and resource costs. When the strength of the association between radiomic features and the outcome is unknown, as generally happens in clinical applications, it is possible to compare results of PR with those of the tree-based methods: if results are similar, we may hypothesize a medium–high association signal. This happened, for instance, in our original study [29] and in the present reanalysis of this NSCLC patient dataset. Indeed, for our previous publication [29] we chose to present the results obtained with PR, which showed similar performances to tree-based methods.

For small datasets (*N* = 100), predictive performances generally decreased and, as expected, they varied within a wide range even with the same classifier. When the association signal was high, the tree-based methods RF and XGBoost still achieved good performances, while PR worked well on small balanced datasets with a low signal. XGBoost accounted better for unbalanced datasets, returning the best performances, and may be indicated as the preferable method for unbalanced, small samples. For simplicity, we collected the suggested methods according to sample, balancing and signal strength in Table 5. Sample size is an important factor, especially in radiomic-based studies. It has been demonstrated that ML methods require at least some hundreds of samples to achieve a low mean-square-error in prediction [33]. Therefore, it would always be preferable to have a medium/large sample size for radiomic studies, especially in cases of low association between radiomic features and outcome. As suggested by our results, in this case it would be difficult to achieve a good model performance and to correctly identify the most important features contributing to the outcome classification.

Previous studies found RF as the best classifier in a similar context of CT lung cancer images [6,26], while others identified other methods, like Naïve Bayes [8], elastic net and SVM [28] as the most accurate. Inconsistent results in the literature are not surprising as they may depend on different sample sizes, unbalanced classes, and on the strength of the association signal. Wu et al. (2016) [8] for instance, compared models trained on an unbalanced dataset of 203 samples (25% squamous carcinoma, 75% adenocarcinoma) and found a slightly lower performance in RF; different from Zhang et al. (2017) [26] who applied SMOTE correction on a dataset of 112 patients. Considering the general low performance results among ML approaches investigated by Wu et al. (2016) [8] and Delzell et al. (2019) [28], a low signal association could be linked to this behavior, where the target variable was different with respect to [6] and [26] in which survival analysis was applied. We were able to quantify the strength of the association signal thanks to data simulation, but it is generally unknown in studies on real data. The large variability of results observed in different studies further highlighted that, as previously reported [6,8,26,27], several aspects should be taken into account when choosing the classifier.

Classical ML algorithms generally assume a balanced representation of samples in each class [34] and are biased towards the majority class when representation is unbalanced. This typically occurs in clinical contexts, where the minority class is often the most important to classify. This makes it extremely important, for unbalanced samples, to evaluate the performance of the classifiers not only in terms of accuracy (AUC), but also in terms of SE and SP. First, we strongly recommend using a resampling technique when using ML approaches in unbalanced samples, especially when high SE is clinically significant. Indeed, we obtained higher values of SE for all the scenarios when the SMOTE correction [32] was applied in comparison to the analysis on non-resampled patients. Regardless of the association signal, RF and XGBoost obtained the best trade-off between SE and SP for medium and large samples, suggesting that these methods are also preferable for unbalanced studies (see Table 5). The importance of accounting for unbalanced data was not pointed out enough, or not even mentioned, in recent radiomic guidelines [35]. Most radiomic studies indeed applied ML techniques and calculated only the accuracy of the prediction, without evaluating their impact on the classification of the less-represented class, even when it is the one of main clinical interest [36]. The correct application of resampling and the choice of the most appropriate ML classifier may partially overcome the impact of unbalanced datasets. It remains the role of clinicians to identify which would be the minimum necessary performance of a classifier, both in terms of SE and SP for each specific context.

The results obtained in this study on the choice of the most suitable classifier indicate that, when datasets are complex as in the radiomic field, more algorithms and more techniques related to ML should be explored before proceeding with the classification: some non-linear ML algorithms, such as tree-based ones, may produce more accurate predictions than logistic or penalized regression, for example if the outcome variable does not depend linearly on other features.

Preselection is strongly recommended in radiomic studies to overcome feature redundancy and high correlation among radiomic data [35]. Nevertheless, we observed that preselection techniques did not generally improve predictive performances on our simulated data when a method that already included an FS step was applied (RF, XGBoost and PR). Moreover, in large–medium scenarios RF, XGBoost, and PR without feature selection were able to retain the three features associated to the outcome, among the 20 most important ones. On the contrary, KNN, and SVM performances increased after preselecting features. Looking at specific FS methods, the lowest performances were observed with the RELF and MRMR methods, especially on samples characterized by a low association with the target. Moreover, RELF had a poor ability to recover the three features associated to the outcome, behavior that could be attributed to the univariate nature of the algorithm, which does account for redundancy among features. PCA (Delta) + WLCX seem to offer a small benefit among other FS methods.

Previous studies investigating the performance of FS methods, identified PCA [26], RELF [8], linear combination or correlation [28,37], and WLCX [6] as the preferable methods in the context of CT lung cancer patients. Only one study [26] applied classifiers without a preselection FS step and obtained comparable results to other FS methods for RF, as observed in our study. Generally, FS methods seemed to have a small impact on the classification performance and their choice had less influence on the model accuracy, as previously pointed out [6,8,25,27]. When we applied FS and classification methods to our original dataset, we found similar performances after preselection techniques compared to no preselection. A possible explanation is that the FS steps may acquire increasing importance with the increase of correlation among radiomic features. Indeed, through our simulation process, we were able to maintain a similar correlation structure with respect to the original one, but with lower overall correlations among the features. In summary, we suggest carefully considering the application of an FS method when using classifiers already including a selection step, and possibly to apply only a small feature reduction to decrease the overall correlation among features. FS should be otherwise mandatory for other classification methods such as KNN and SVM.

Recently published studies [38,39] highlighted the need for safeguards in radiomics analysis, such as checking for multicollinearity and volume-confounding effects. Our classification framework may be integrated with other preprocessing steps to take into account adjustments for clinical variables, including the tumor volume, and other safeguard analyses, as applications of harmonization methods [40,41,42].

Other clinical variables, such as gender, side and sites may affect the outcomes of cancer patients and should be taken into account in the analysis of clinical studies, as we did in our previous clinical publication on these patients [29]. Moreover, as explorative analysis, we investigated whether significant differences in these relevant clinical variables were presented among radiomic features. In particular our data showed that, among the three features simulated to be highly associated with the lymph node status, only F3 was significantly different according to gender and site.

Strengths of our study include the wide numbers of scenarios considered for evaluating the choice of FS and classifiers in different contexts, that would not be feasible when a single dataset is considered. Moreover, with simulated data we knew *a priori* which features were associated to the outcome with a predefined low or high signal. To our knowledge this gave us the opportunity to evaluate for the first time, not only the accuracy of the models in terms of AUC, but also to identify which approaches were most able to correctly identify the true associations, thus overcoming model overfitting. As reported by Zhovannik et al. [43], radiomic features may depend on image acquisition settings, and therefore variations in the features may cause a reduction in prediction performance. A preliminary selection with ANOVA was performed on our original dataset [29] to choose features that were not significantly affected by the use of different scanners and reconstruction algorithms. Indeed, we applied our analysis on homogeneous data, by selecting the 168 radiomic features that resulted as independent from the abovementioned parameters (see our previous study in [29]). Thus, the introduction of uncontrolled sources of variability, that may be present in other studies with a heterogeneous acquisition of radiomic data, can be avoided.

Finally, among limitations, our simulated data were generated by a specific dataset of CT images of early-stage NSCLC patients, which may partially reduce the reproducibility of the results for other imaging techniques and tumor types. Further studies are suitable to investigate whether similar recommendations could be applicable in other contexts. Second, both patients from the training and validation sets were simulated by the same sample of patients and may therefore be more similar to each other than would be expected in external datasets. For this reason, our performance results on the validation set may be generally higher than they would be if external validation were considered. However, as this consideration applies to all the scenarios, FS and classification methods, it does not affect the comparison among the various approaches and the recommendations arising from our study.

As a final consideration, it must be highlighted that the present NSCLC dataset was adequate for the purpose of deriving a useful indication for radiomic studies in this particular clinical scenario and, more importantly, of implementing and refining a methodology applicable to different radiomic databases and clinical endpoints. In this regard, to improve clinical significance for the specific early-stage NSCLC pathology investigated here, the analysis could be extended to also include radiomic features extracted from PET images in addition to those obtained from CT images. Promising results were obtained from previous studies investigating the prognostic role of PET and PET/CT radiomic features in a NSCLC population undergoing surgery [44], as well as in locally advanced NSCLC disease [45] and pleomorphic lung carcinoma [46]. Similarly, PET/CT radiomic features could play a role in the molecular characterization of lung pathology [47]. More in general, the integration of multiple -omics data originating from different image modalities and other clinical examinations appears to be the proper way towards clinical implementation of personalized medicine [48], a scenario which cannot exclude the investigation of the optimal way to implement and fully exploit the potentialities of artificial intelligence approaches.

## 5. Conclusions

Our analysis showed that the choice of FS and classification methods in radiomic studies can consistently influence the results.

We strongly suggest carefully evaluating their choice, taking into account the specific sample size, balancing (balanced/unbalanced), and the hypothesized strength of the association. The tree-based methods RF and XGBoost generally obtained the best performances in all scenarios, but PR may be preferable for medium/large samples with a high signal due to its easier interpretability and computational simplicity. FS methods may be generally avoided when RF, XGBoost, and PR methods are used, while they should be used before applying KNN and SVM classifiers.

## Figures and Tables

**Table 1:** Summary of the simulated samples included in the analysis as combinations of three sample sizes (*N* = 600, 300 and 100), balanced/unbalanced and high/low signal *.

**Sample Size**	**Balanced Samples** **(50% pN = 0, 50% pN = 1)**						
	FBP		IR		Total (test + validation set)		
	PN0	PN1	PN0	PN1	PN0	PN1	All PN
Large	200	200	100	100	300	300	600
Medium	100	100	50	50	150	150	300
Small	34	33	16	17	50	50	100
**Sample Size**	**Unbalanced Samples** **(70% pN = 0, 30% pN = 1)**						
Large	280	120	140	60	420	180	600
Medium	140	60	70	30	210	90	300
Small	47	20	23	10	70	30	100

FBP = Filtered Back Projection; IR = Iterative Reconstruction; PN0 = no pathological node; PN1 = any pathological node. * The six samples here described are replicated with the same numbers for the two hypotheses of high signal and low signal, giving a total number of twelve simulated samples included in the analysis.

**Table 2:** List of applied feature selection methods (FS) and classifiers with acronyms used in the text.

FS Acronym	FS Method	Classifier Acronym	Classifier
No FS	No feature selection step	PR	Penalized Regression
HC + WLCX	Hierarchical clustering + Wilcoxon	RF	Random Forest
PCA (Delta) + WLCX	Principal Component Analysis clustering with Delta plot stop criterion + Wilcoxon	XGBoost	Extreme Gradient Boosting
PCA (Proportion) + WLCX	Principal Component Analysis clustering with proportion stop criterion + Wilcoxon	LSR	Logistic Step-wise Regression
MRMR	Minimum Redundancy Maximum Relevance	KNN	K-Nearest Neighbor
RELF	Relief	SVM	Support Vector Machine

**Table 3:** Mean AUC for each classifier across different sample sizes, balancing, signal intensity and feature selection methods in the validation set. Best mean AUC, Sensitivity and Specificity values are in bold.

Method	AUC Mean	AUC SD	Sensitivity Mean	Sensitivity SD	Specificity Mean	Specificity SD
Classification methods						
KNN	0.63	0.12	0.43	0.21	0.65	0.21
LSR	0.64	0.11	0.45	0.22	0.74	0.14
PR	0.67	0.12	0.45	0.24	0.78	0.15
RF	0.78	0.16	0.58	0.24	**0.82**	0.15
SVM	0.63	0.12	0.44	0.29	0.68	0.3
XGBoost	**0.79**	0.16	**0.62**	0.23	0.81	0.13
Feature selection methods						
No FS	**0.72**	0.12	**0.56**	0.24	**0.76**	0.15
HC + WLCX	0.71	0.12	0.54	0.21	0.74	0.21
MRMR	0.63	0.11	0.41	0.22	0.73	0.14
PCA (Delta) + WLCX	**0.72**	0.16	0.51	0.24	**0.76**	0.15
PCA (Proportion) + WLCX	0.71	0.12	0.52	0.29	**0.76**	0.30
RELF	**0.67**	**0.16**	**0.44**	**0.23**	**0.74**	**0.13**

AUC = Area Under the receiver operating characteristics Curve; SD = Standard Deviation; KNN = K-Nearest Neighbor; LSR = Logistic Step-wise Regression; PR = Penalized Regression; RF = Random Forest; SVM = Support Vector Machine; XGBoost = Extreme Gradient Boosting; FS = Feature Selection; HC + WLCX = Hierarchical clustering + Wilcoxon; MRMR = Minimum Redundancy Maximum Relevance; PCA (Delta) + WLCX = Principal Component Analysis clustering with Delta plot stop criterion + Wilcoxon; PCA (Proportion) + WLCX = Principal Component Analysis clustering with proportion stop criterion +Wilcoxon; RELF = Relief.

**Table 4:** Discriminative performance of combinations of feature selection and classification methods applied to real data.

Feature Selection	Classification	AUC (CI 95%)	Sensitivity	Specificity
No FS	PR	0.60 (0.46, 0.73)	0.50	0.61
	RF	0.62 (0.49, 0.75)	0.50	0.63
	XGBoost	0.64 (0.51, 0.76)	0.50	0.70
	KNN	0.57 (0.45, 0.67)	0.54	0.59
	SVM	0.52 (0.38, 0.65)	0.50	0.57
HC + WLCX	PR	0.63 (0.50, 0.76)	0.46	0.63
	RF	0.67 (0.54, 0.79)	0.50	0.70
	XGBoost	0.64 (0.51, 0.76)	0.54	0.65
	LSR	0.57 (0.43, 0.71)	0.50	0.61
	KNN	0.55 (0.44, 0.67)	0.37	0.73
	SVM	0.55 (0.42, 0.68)	0.62	0.59
PCA (Delta) + WLCX	PR	0.60 (0.47, 0.72)	0.58	0.63
	RF	0.65 (0.52, 0.78)	0.67	0.61
****	XGBoost	0.69 (0.57, 0.8)	0.67	0.62
****	LSR	0.59 (0.46, 0.72)	0.50	0.64
****	KNN	0.58 (0.45, 0.70)	0.54	0.59
****	SVM	0.58 (0.45, 0.70)	0.62	0.57
PCA (Proportion) + WLCX	PR	0.57 (0.44, 0.71)	0.42	0.65
	RF	0.64 (0.50, 0.77)	0.54	0.65
	XGBoost	0.69 (0.55, 0.81)	0.54	0.65
	LSR	0.58 (0.43, 0.72)	0.50	0.68
	KNN	0.58 (0.46, 0.69)	0.45	0.70
	SVM	0.60 (0.48, 0.72)	0.66	0.59
MRMR	PR	0.55 (0.42, 0.68)	0.42	0.63
	RF	0.64 (0.50, 0.77)	0.46	0.72
	XGBoost	0.66 (0.52, 0.78)	0.54	0.65
	LSR	0.54 (0.40, 0.67)	0.42	0.68
	KNN	0.50 (0.39, 0.61)	0.33	0.67
	SVM	0.61 (0.48, 0.73)	0.62	0.63
RELF	PR	0.70 (0.57, 0.83)	0.21	0.94
	RF	0.60 (0.47, 0.73)	0.50	0.69
	XGBoost	0.67 (0.53, 0.79)	0.46	0.79
	LSR	0.69 (0.55, 0.82)	0.62	0.69
	KNN	0.45 (0.35, 0.56)	0.29	0.61
	SVM	0.75 (0.63, 0.86)	0.54	0.87

AUC = Area Under the receiver operating characteristics Curve; CI = Confidence Intervals; HC + WLCX = Hierarchical clustering + Wilcoxon; KNN = K-Nearest Neighbor; LSR = Logistic Step-wise Regression; MRMR = Minimum Redundancy Maximum Relevance; PCA (Delta) + WLCX = Principal Component Analysis clustering with Delta plot stop criterion + Wilcoxon; PCA (Proportion) + WLCX = Principal Component Analysis clustering with proportion stop criterion + Wilcoxon; PR = Penalized Regression; RF = Random Forest; RELF = Relief; XGBoost = Extreme Gradient Boosting; SVM = Support Vector Machine.

**Table 5:** Suggested guidelines for the choice of the classifier, according to sample size, balancing and association strength.

Sample Size	Balancing	Association	
		High	Low
Large (600)	Balanced Unbalanced	PR PR	RF * RF *
Medium (300)	Balanced Unbalanced	PR PR	RF * RF *
Small (100)	Balanced Unbalanced	RF * XGBoost	PR XGBoost

* Preferred over XGBoost for lower computational time and less hyperparameters to be tuned.

## Data Availability

The data presented in this study are available on request from the corresponding author. The data are not publicly available because a specific authorization was not obtained by the Institutional Ethics Committee.

## Supplementary Materials

The following are available online at https://www.mdpi.com/article/10.3390/cancers13123088/s1, Supplementary Methods, Figure S1: Classification procedure flowchart, Figure S2: Correlation among radiomic features, Figure S3: Number of features correctly selected as associated with the outcome, Figure S4: Sensitivity and specificity for unbalanced datasets with and without SMOTE correction for large dataset, Figure S5: Sensitivity and specificity for unbalanced datasets with and without SMOTE correction for medium dataset, Figure S6: Sensitivity and specificity for unbalanced datasets with a without SMOTE correction for small dataset, Figure S7: Heatmap representing the predictive performance (AUC) in the validation set for unbalanced SMOTE samples, Table S1: Baseline characteristics of the original study population, Table S2: Wilcoxon *p*-values for the association of features F1, F2 and F3 with outcome. References [29,30,31,32,49,50,51] are cited in the supplementary materials
