# Supplementary material for: The Challenge of Choosing the Best Classification Method in Radiomic Analyses: Recommendations and Applications to Lung Cancer CT Images
Source: Cancers (Basel). 2021 Jun 21;13(12):3088. doi: 10.3390/cancers13123088 (PMC8234634; doi:10.3390/cancers13123088)
Supplement: Supplementary file 1 [file cancers-13-03088-s001.zip › cancers-1186771-supplementary.pdf]

Supplementary Materials:

The Challenge of Choosing the Best Classification Method in Radiomic Analyses: Recommendations and Applications to Lung Cancer CT Images

Federica Corso, Giulia Tini, Giuliana Lo Presti, Noemi Garau, Simone Pietro De Angelis, Federica Bellerba, Lisa Rinaldi, Francesca Botta, Stefania Rizzo, Daniela Origgi, Chiara Paganelli, Marta Cremonesi, Cristiano Rampinelli, Massimo Bellomi, Luca Mazzarella, Pier Giuseppe Pelicci, Sara Gandini and Sara Raimondi

1. Supplementary Methods

1.1. Radiomic features simulation

We first analysed the distribution of the 168 FBP- and IR- derived features in the original Non-Small-Cell Lung Cancer (NSCLC) dataset [1]. We analysed them separately for both the outcome classes to assess their normality (Shapiro's test, p-value < 0.05) and the ranges they cover. Additionally, we computed Pearson's correlation among features. We observed that only 35 over the168 FBP-derived features (21%) and 72 over the 168 IR-derived features (43%) were normally distributed. This fact implies not only difficulties in identifying known distributions, but also differences in the feature values computed by the two CT reconstruction algorithms. High correlation was identified among the features (Supplementary Figure S2).

Then, taking advantage of the real data on NSCLC patients recruited for the previous study [1], we simulated 168 radiomic features to build synthetic controlled scenarios. We could not simulate radiomic features from scratch because of the high intra-feature correlation that characterizes radiomic data and the impossibility to identify known distribution functions for large part of the features. However, both feature distributions and correlation should be maintained also in the simulated dataset.

Our simulation procedure is thus divided in two main steps (Figure 1):

1. simulation of 168 features without association to the outcome
2. selection of balancing and signal level and association of the simulated features and samples to the outcome. We applied both procedures separately to FBP and IR-derived features.

In the first step, we simulated 168 multivariate non normal distributions starting from correlation matrix, skewness and kurtosis of the real NSCLC features as suggested by Vale and Maurelli [2]. We used the R package "fungible" but we modified the provided function monte1 to allow the computation of Cholesky decomposition (parameter pivot=TRUE) also for positive semi-definite matrices. Additionally, the default final scaling to normalize the distribution of the simulated variables was removed. Since the ranges of the radiomic features may vary a lot, we moved the obtained variables to their original ranges. Thus, for each feature Fi distributed on the range $[ai, bi]$ in the original dataset, the intermediate simulated distribution f_i^* was translated and the final distribution was obtained as

$$f_i = \frac{(b_i - a_i)[f_i^* - \min(f_i^*)]}{[\max(f_i^*) - \min(f_i^*)]} + a_i \tag{1}$$

In the second main step of the simulation procedure, we associated samples and features to different outcome classes and we introduced the possibility to create balanced/unbalanced datasets with features carrying high or low signal.

First, we chose the sample balancing according to an equal (balanced) or unequal (unbalanced) distribution of the outcome classes. In the former case we randomly assigned 50% of the samples to the class pN=1, in the latter only 30%. Two preliminary sets of data, each one consisting in 600 samples, were therefore created.

We then associated features to the outcome, to generate the signal able to separate samples with different outcome class. We chose features found to be significantly associated with the positive lymph node in the original dataset[1]: ClusterShade from GLCM25 category calculated along 135° direction with four voxels offset (F1), 70th percentile of the intensity values in the cumulative histogram (F2), and the maximum diameter evaluated on the 3D lesion volume (F3).

If we indicate with $f_i,\ \ i \in \{1,2,3\}$ the simulated features obtained at step 1 from the selected ones and with $mean(fi)$ their mean value over samples, then the association to the outcome was obtained by translation of f_i by a factor dependent on the mean:

$$sf_{i,\{0,1\}} = f_i \pm \frac{mean(f_i)}{k_i},\ i \in \{1,2,3\} \quad (2)$$

The direction of the translation was assigned according to original data: the distribution for the positive class $sf_{i,1}$ was obtained by addition and the distribution for the negative class $sf_{i,0}$ by subtraction whenever the original feature distribution for pN=1 had larger mean than pN=0.

The factors ki were selected specifically to obtain simulated features highly (high signal) or poorly (low signal) associated to the outcome: in particular, they were chosen so that the distributions $sf_{i,1}$ and $sf_{i,0}$ could be well or poorly separated on the bases of Wilcoxon test.

Finally, to better resemble real radiomics data, we added gaussian noise to our simulated data.

Four datasets including 600 simulated patients each were eventually obtained, according to the combination of balancing (balanced/unbalanced) and signal (high/low) (Figure 1).

1.2. Description of classifiers and feature selection methods

For each of the 12 simulated scenarios, six different machine learning methods, combined with five feature selection methods or no feature selection step, were investigated (Table 2). For each scenario, feature selection methods and classifiers were trained on the training set and then tested in the validation set.

1.2.1. Feature selection methods

The purpose of feature reduction is to select a subset of uncorrelated and useful features in order to improve the prediction accuracy of the models. Feature selection methods are usually grouped into three categories: filter methods, wrapper methods and embedded methods. In the present work, only filter methods were considered since the selection process was independent by the classifier.

Five different supervised feature selection methods were investigated in our analysis.

Three of them consist in the combination of a dimensionality reduction method with Wilcoxon Rank Sum test. Specifically, the following two steps were applied:

1. partition of the original set of features (168) into K clusters, using one among the following procedures: Hierarchical, Hierachical+PCA based on Delta plot, Hierarchical+PCA based on proportion of explained variance (details below).
2. use of Wilcoxon Rank Sum test to identify, in each cluster, the feature with the highest correlation with the outcome and select the K most predictive features. In the three dimensionality reduction methods, the number of K cluster was established during

the clustering procedure. Step 1 allows therefore to group redundant features while step 2 acts as univariate features filter.

The last two feature selection methods are filter-based methods consisting in a single step and are known as Relief and minimal Redundancy Maximum Relevance (MRMR) respectively. Both algorithms require the computation of a score through which feature importance is established. According to the scoring criteria, Relief is considered a univariate method since the importance of each feature is established without taking into account relations between the other predictive variables. Conversely, MRMR can be considered as a multivariate selection method. Further details about the scoring procedure are given in the following for the two strategies.

Details of each of the five methods are reported here:

1. *Hierarchical.* The generic hierarchical procedure was constructed implementing an in-house function to identify clusters of radiomic features highly correlated with each other. In particular, we considered the Spearman correlation and included in the same cluster radiomic features with a correlation ≥0.75.
2. *Hierachical+PCA based on Delta plot*. This procedure is based on dimensionality reduction and it is implemented in the software R using the CLV function in the package "ClustVarLV" [3]. The CLV approach is based on the construction, within each cluster, of a latent variable obtained as a linear combination of only the variables belonging to the corresponding cluster. This was done maximizing the following criterion:

$$T = \sum_{k=1}^{K} \sum_{j=1}^{p} \delta_{kj} cov^2 r\ (x_j, c_k) \qquad with\ var(c_k) = 1 \tag{3}$$

were $x_j (j = 1, \ldots, p)$ are the p variables to be clustered, K is the number of clusters, c_k (k=1, ..., K) is the latent variable associated with the kth cluster and δ_{kj} reflects a simple membership, with $\delta_{kj} = 1$ if the jth variable belongs to the kth cluster and $\delta_{kj} = 0$ otherwise. This criterion characterizes the so-called directional method, in which, regardless of a positive or negative correlation, the latent variable is constructed so that its correlation to the observed variables is as high as possible. In CLV approach, the choice of the number of clusters K is made based on an evaluation of the Delta plot, where $Delta = T(k) - T(k-1)$. In fact, when the value of Delta clearly jumps, it means that there is an important loss in homogeneity of the clusters when passing from K to $K - 1$ clusters, thus preferring K clusters.

3. *Hierarchical + PCA based on proportion of explained variance.* We performed this method by using SAS Software, VARCLUS procedure. The maximization criterion is similar to the above mentioned for CLV function, but we chose here a different stop criterion in clusters generation. Specifically, we established as 0.80 the proportion of explained variance by the latent variable, as previously performed [4,5], so that the number of clusters was automatically determined based on the capacity to maintain this proportion in each cluster.
4. *Relief.* Relief algorithm is based on the concept that good attributes are those with similar values among instances of the same class and different values among instances of different classes. To quantify the importance of a feature, a weight is established looking at the neighbors of each instance. Specifically, given an instance and its neighbors, the score of a feature is derived subtracting the distance if the neighbor belongs to the same class and adding the distance for neighbors of a different class. Following, the equation applied to compute the Relief score of a feature X_k is reported:

$$J_{Relief}(X_k) = \frac{1}{2} \sum_{t=1}^{p} d\big(X_{t,k} - X_{NM(x_t),k}\big) - d(X_{t,k} - X_{NH(x_t),k}) \tag{4}$$

were $X_{t,k}$ is the value of instance x_t on feature X_k. $X_{NM(x_t),k}$ and $X_{NH(x_t),k}$ are the values on the k'th feature of the nearest point to x_t with the same and different class label respectively, and de denotes the distance.

5. *mRMR.* The MRMR algorithm relies on the combination of two constraints. Maximum-Relevance (MR) is the first constrain and consists in the maximization of the relevance given by the mean value of all mutual information values between individual features x_i of the feature set S and class c as reported in the following equation:

$$\max D(S,c), D = \frac{1}{|S|}\sum_{x_i \in S} I(x_i; c) \quad (5)$$

Since, according to MR, a high dependency among features can be reached, the second constrain aims to minimize the redundancy (Minimal Redundancy, mR). To find mutually exclusive features, the minimization of redundancy is applied as follows:

$$\min\ R(S), R = \frac{1}{|S|^2}\sum_{x_i, x_j \in S} I(x_i, x_j) \quad (6)$$

According to MRMR, the best features correspond to those with highest difference between the two constrains.

1.2.2. Classification methods

We assessed the predictive performances of six machine learning classifiers (Table 2): Penalized Regression methods (PR), Logistic step-wise Regression model (LSR), Random Forest (RF), Extreme Gradient Boosting (XGBoost), K-Nearest Neighbor (KNN) and Support Vector Machine (SVM).

Penalized regression (PR). Penalized regression is based on a simple linear regression model, where a certain set of independent variables is used to predict the so-called dependent variable, which can be of different types. The penalization concerns the model's coefficients, and it is used to reduce their variance, without a substantial increase in bias. Two regularization parameters can be optimized: α and λ. The first parameter (α) defines the type of penalization to be applied and it can assume values from 0 (Ridge regression) to 1 (Lasso regression). When α=1 the penalization can be intended as operating a features selection because it forces the coefficients near to 0 to exactly be equal to 0. The second parameter (λ) regulates the penalization strength. It can assume values from 0 (no penalization) to infinite, with larger values corresponding to stronger penalizations. We optimized the two parameters using "cv.glmnet" in "glmnet" R package and performed the penalized regression specifying "glmnet" in "Caret" R package [6].

Random Forest (RF). Random Forest is a supervised machine learning method created by the aggregation of a certain number of classification trees using the bagging approach. The basic idea of this method is that the combination of many uncorrelated trees will reduce the final overfitting and the predicted values are obtained calculating the mean of the predicted values from each tree. In Random Forest, several parameters must be set: the number of trees to be aggregated, the number of predictors to randomly include in each node and the minimum number of nodes to use in each tree. In this paper, Random Forest has been performed using the "ranger" method in "caret" R package [6], thus the first parameter has been set to 500 by the "ranger" default, while for the others a grid of values has been created and the values leading to the best performance (based on AUC) have been used. The same approach has been used to detect which nodes splitting rule gives the best performance among the methods available in "ranger", that is "Gini", "Extratrees" and "Hellinger".

Logistic step-wise regression model (LSR). Logistic step-wise regression model belongs to the linear regression model family in which the dependent variable (also called response variable) is binary. The output of the model is the probability that a certain event occurs, which can be then treated as a dichotomous variable to classify the outcome. In order to avoid convergence problem, logistic regression model was always coupled with a feature selection method prior to modelling. Moreover, in case of many variables selected (some dozen) and medium-small sample size, only features with p-value ≤ .15 were maintained for modelling. In particular, a step-wise technique was adopted, as further step of selection, to fit the model. In each step, a variable was added or subtracted from

the set of explanatory variables based on the chosen criterion (p-value ≤ .15). Akaike Information Criterion (AIC) was used as measure to select the final model. AIC measures goodness of fit, but it also includes, in its formula, a penalty that increases as the number of the estimated parameters gets bigger. The logistic step-wise regression model was performed specifying the method "glmStepAIC" in "caret" R package [6].

Extreme Gradient Boosting (XGBoost). Extreme Gradient Boosting adopts the boosting statistical technique in which a predictive model is performed in the form of an ensemble of weak predictive models. This is achieved by resampling data and giving more weight to those which are misclassified. In this way, a new classifier that would boost the performance is computed. This process is repeated, generating a set of classifiers, which are ultimately combined through voting to define the final classifier. Here, decision trees were chosen as weak learners and squared error was used as loss function. Each parameter was tuned step by step upon a well-defined search grid (for more details about tuning parameters see "xgboost" R package). The Extreme Gradient Boosting model was performed specifying the method "xgbtree" in "caret" R package [6].

Support vector machine (SVM). Support vector machine is a supervised machine learning algorithm which aims in finding decision boundaries that better separates instances of different classes. The best boundaries correspond to those which maximize distance from training instances (larger margin) and minimize misclassification. The "support vectors" are represented by the training set data points closest to the boundaries.

One of the most important parameters to be optimized is the parameter "C" which determines margin width: a lower margin (larger C) brings to a better separation of training instances but lacks in generalization ability, while with larger margins (lower C) a better generalization of the model should be guaranteed at the expense of a lower accuracy in classifying training samples. The other parameters to be optimized are usually dependent on the adopted kernel which determines how the data are mapped in a new multidimensional space. Choosing the proper kernel type allows to transform data making them linearly separable.

In the presented work we considered a Radial Basis Function (RBF) kernel which transforms data through a Gaussian function. The parameter to be optimized in this case is which determines the variance of the Gaussian. A small gamma implies the class of this support vector will have influence on deciding the class of vector. If gamma is large then variance is small implying the support vector does not have wide-spread influence. To test the RBF SVM with RBF kernel, the function "svmradial" was adopted. Using caret package pipeline [6], parameters C and gamma were optimized through a grid search investigating values in the logarithmic range 0.1-10 and 0.001-1, respectively.

K nearest-neighbors (KNN). K nearest-neighbors is a supervised algorithm based on the concept that similar instances belong to the same class. The main parameter in this algorithm is the K which defines the number of neighbors to which the instance needs to be compared to estimate its class.

The level of similarity is usually calculated through a distance function. Considering the class of the k nearest instances, the predicted class will be determined through a majority voting which can be also weighted according to their distance from the predicted variable. K is a parameter which needs to be optimized since a K to small make the classifier sensitive to noise while a K too large will cause the inclusion of more samples belonging to the other class. To test the KNN algorithm the R function "kknn" of "caret" R package [6] was adopted. After preliminary tests, a "triangular" kernel was set to weight the neighbours according to their distances while the K parameter was optimized searching between a set of odd values (3-47) to avoid parity conditions.

1.3. Detailed classification framework

Commonly to all machine learning studies, we designed a systematic classification framework which consists of canonical steps including: pre-processing, cross-validation, performance evaluation and classification. Finally, we characterized the model with the

optimal prediction metrics on the validation set and we analysed the stability of each machine learning method across the different scenarios. The main steps of the classification framework are visualized in Supplementary Figure 1 and are described below:

1. *Pre-processing*: we randomly selected 2/3 of the patients as training set and the remaining 1/3 as validation set. We used the same random split all over the models. In case of feature selection, the algorithm was applied on the training set (Supplementary Figure 1) and consequently only the selected features were maintained in the validation set. Pre-processing as centering, scaling and non-zero-variance were applied to the entire datasets.
2. *Cross-validation (CV):* 10-fold cross validation was applied to the training set for each considered classifier. At each CV round, the classifier was trained using nine folds and evaluated by AUC on the 10th fold as test set. The hyperparameters tuning was computed on a well-defined search grid. After repeating the process all over the folds, predictions were compared with true labels, adopting the AUC as evaluation metric. This CV procedure was repeated three times (3X10 CV) in order to improve the accuracy of the parameters estimation and to reduce over-fitting. In addition, a resampling method was applied to the unbalanced sample. In particular, the Synthetic Minority Over-sampling (SMOTE) technique was chosen, as it has been shown to give consistent results with respect to dataset having different unbalanced ratio [7]. SMOTE is an iterative method of over-sampling the minority class, in which new samplings are synthesized according to the closest minority neighbor (Euclidean distance), thus resulting in an augmented proportion of the minority class while maintaining the same original dataset structure.
3. *Final model definition:* to determine the best parameters among those investigated in the 3X10 CV, Area Under the ROC Curve (AUC) was used. The final model was then derived training the classifier on the entire training set and setting as hyperparameters those found to be the best.
4. *Predictive performance evaluation:* the performance of the final classifier was then evaluated on the test set through AUC, along with sensitivity and specificity.

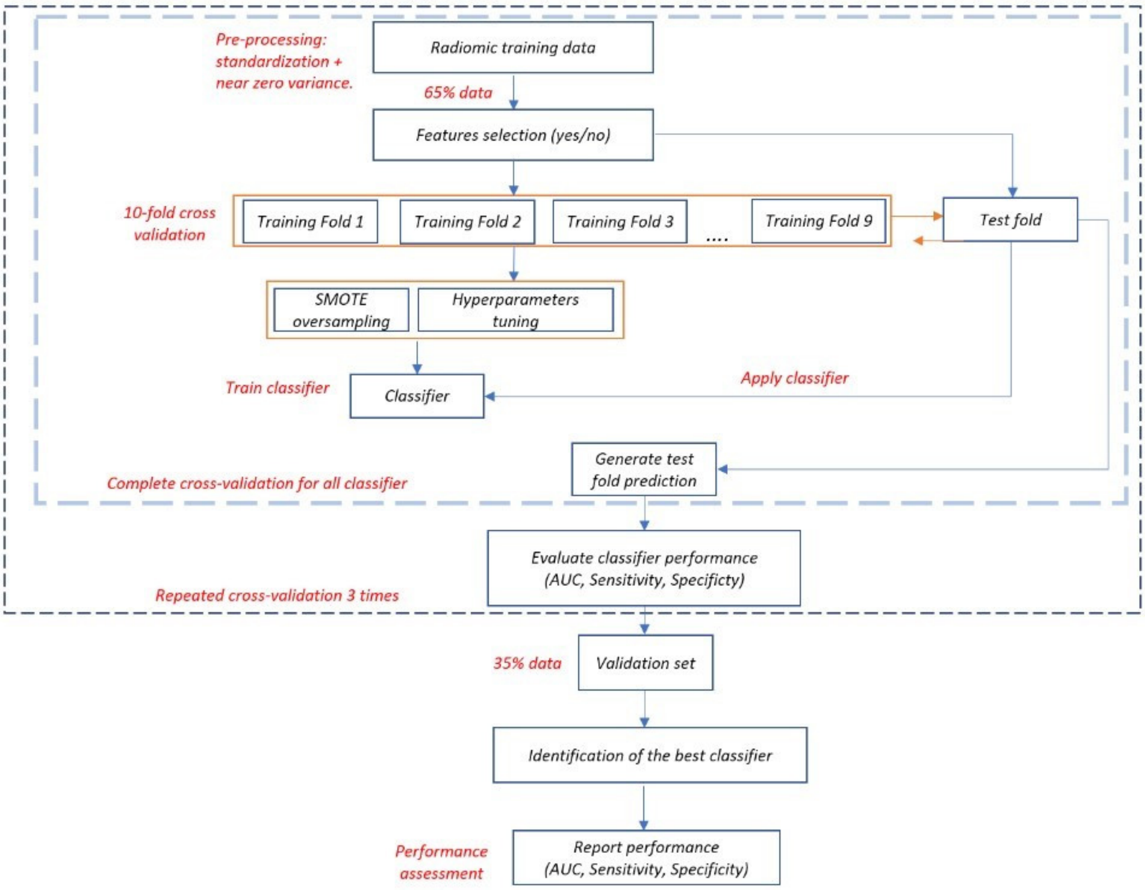

Figure S1. Classification procedure flow chart. After pre-processing of radiomic data, each dataset was split into training (2/3 of the subjects) and validation (1/3) set. After possible application of different methods of features selection, class imbalance correction for unbalanced datasets, and hyperparameters tuning, classification methods were applied and their performance evaluated by the Area Under the Receiver Operating Characteristics Curve (AUC), Sensitivity and Specificity both in the training and in the validation set. The best classifiers were identified as the ones with the best performance in the validation set. .

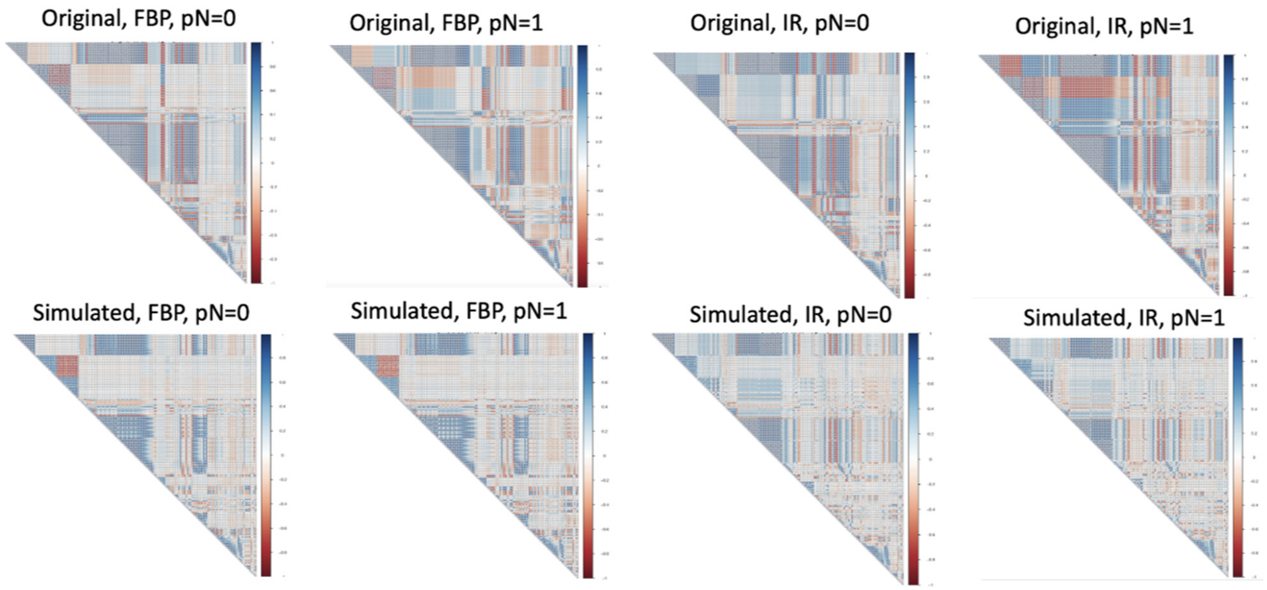

Figure S2. Correlation among radiomic features. Correlation matrices are reported for the original NSCLC dataset (upper panel) and for the simulated case (lower panel) by algorithm and outcome variable. Intensity of colours represent the level of correlation, with blue shades used for positive and red shades for negative correlation.

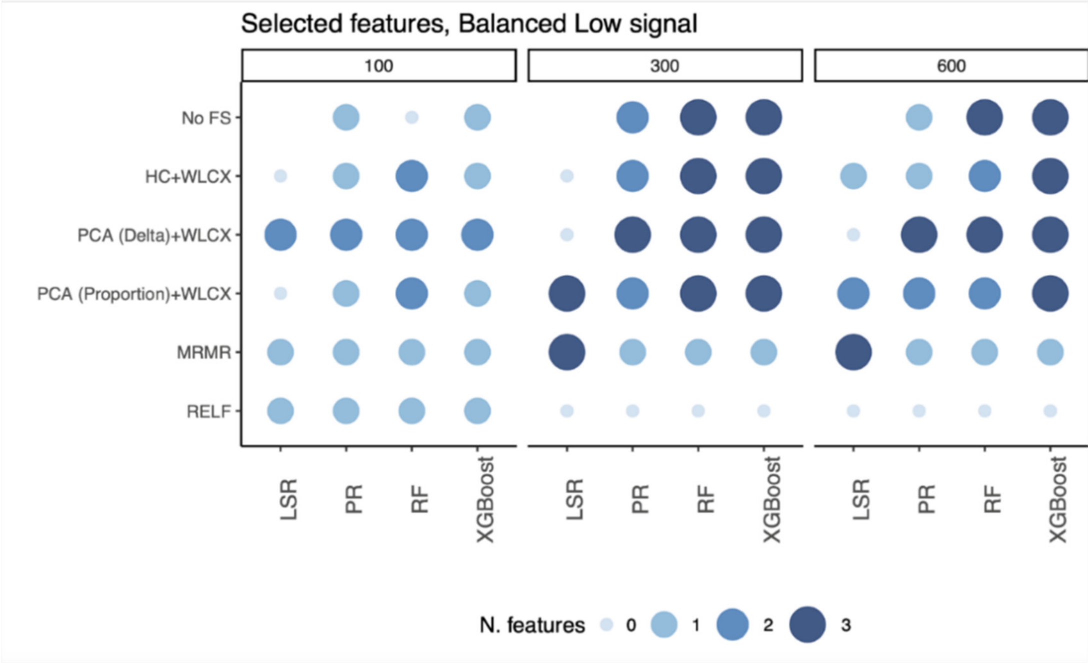

A)

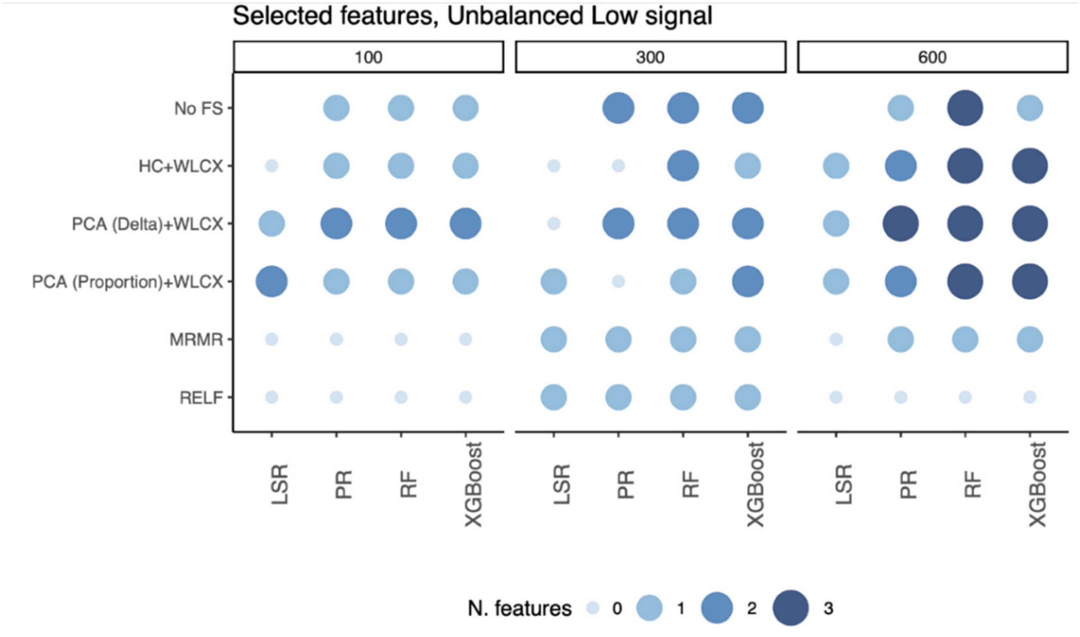

B)

Figure S3. Number of features correctly selected as associated with the outcome. Results for four classification methods for balanced (panel A) and unbalanced (panel B) dataset are reported. According to our study design, three features (namely F1, F2 and F3) have been simulated as to be associated with the clinical outcome. The plot reported the number of features among these three that were indeed correctly selected among the top 20 by the four classification methods for which a selection or importance values could be obtained.

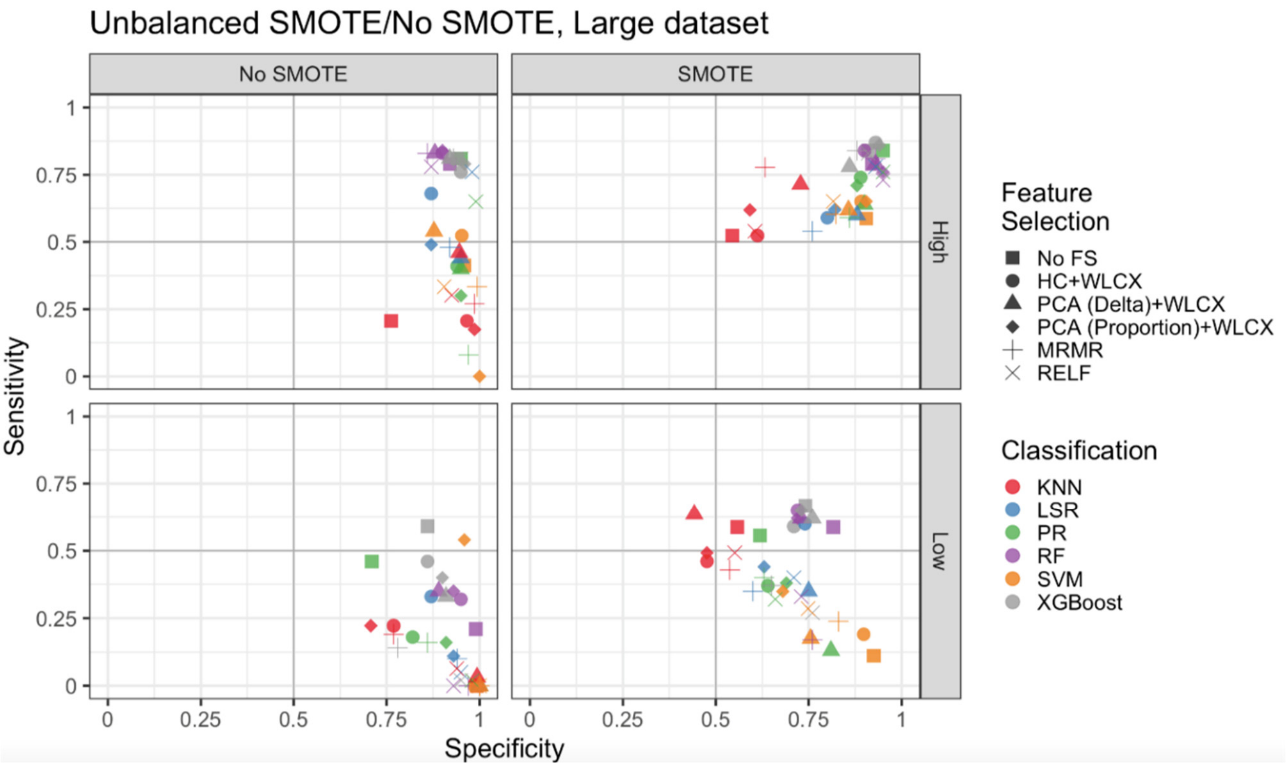

Figure S4. Sensitivity and specificity for unbalanced datasets with and without SMOTE correction for large dataset. Performances of the Machine Learning (ML) algorithms and feature selection methods applied to the unbalanced cases are displayed for high signal (upper panels) and low signal (lower panels). Columns report results for analysis without (left) or with (right) application of smote adjustment. Colours are used to distinguish ML algorithms, shapes for feature selection methods.

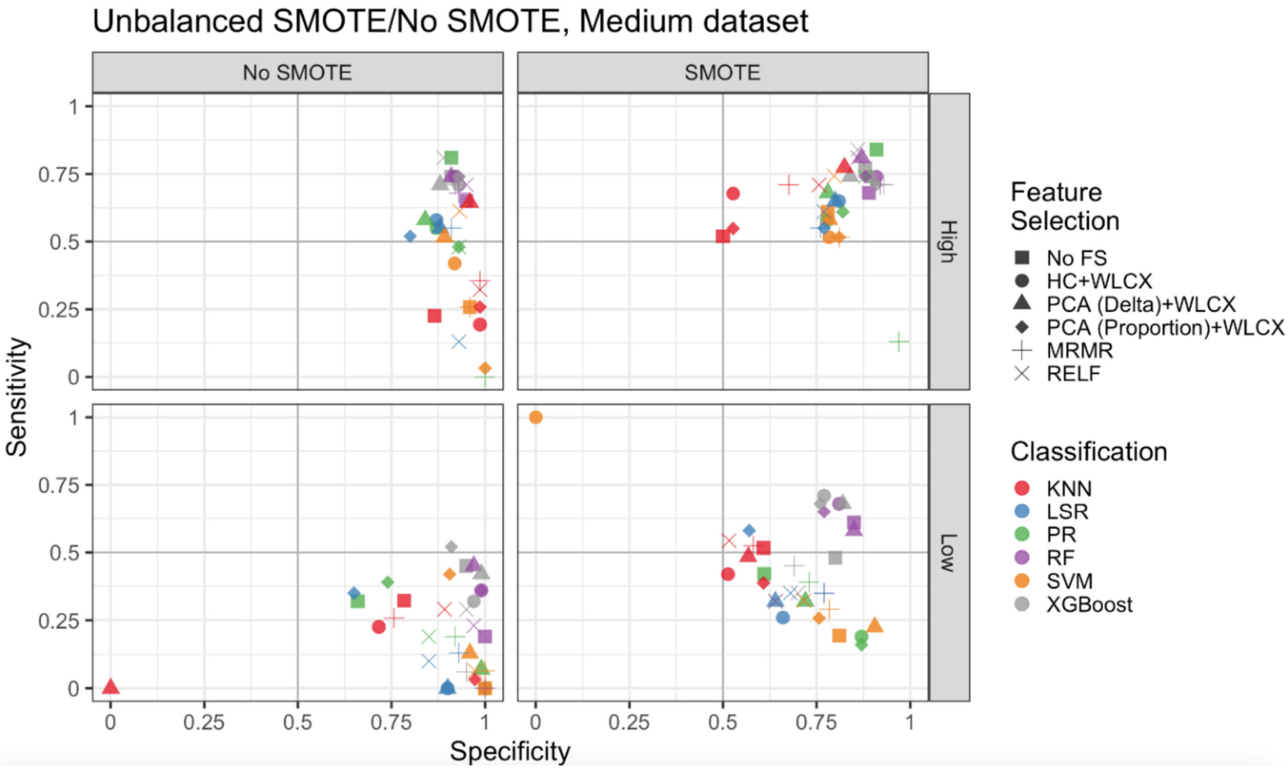

Figure S5. Sensitivity and specificity for unbalanced datasets with and without SMOTE correction for medium dataset. Performances of the Machine Learning (ML) algorithms and feature selection methods applied to the unbalanced cases are displayed for high signal (upper panels) and low signal (lower panels). Columns report results for analysis without (left) or with (right) application of smote adjustment. Colours are used to distinguish ML algorithms, shapes for feature selection methods.

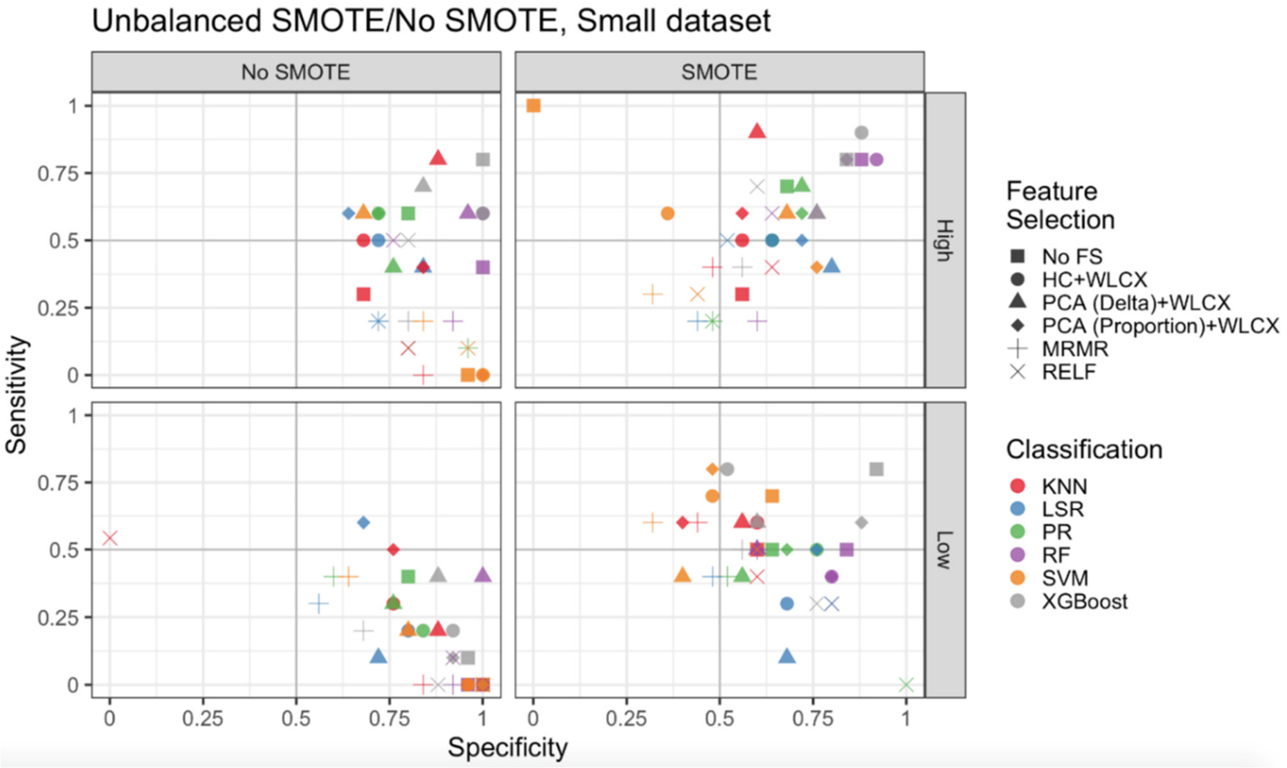

Figure S6. Sensitivity and specificity for unbalanced datasets with and without SMOTE correction for small dataset. Performances of the Machine Learning (ML) algorithms and feature selection methods applied to the unbalanced cases are displayed for high signal (upper panels) and low signal (lower panels). Columns report results for analysis without (left) or with (right) application of smote adjustment. Colours are used to distinguish ML algorithms, shapes for feature selection methods.

A) **B)**

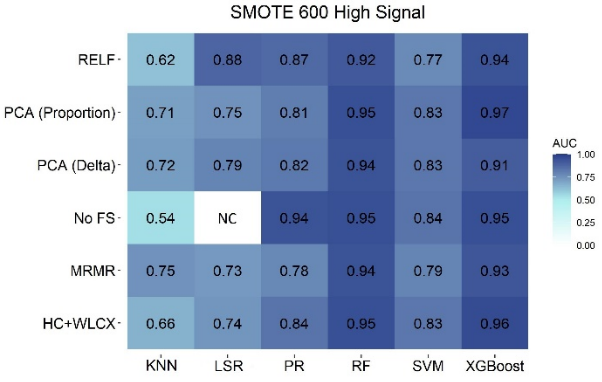

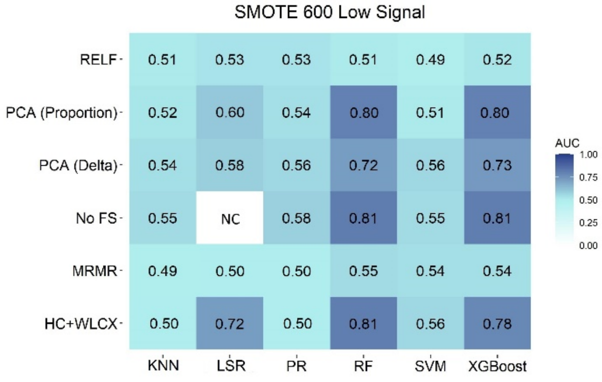

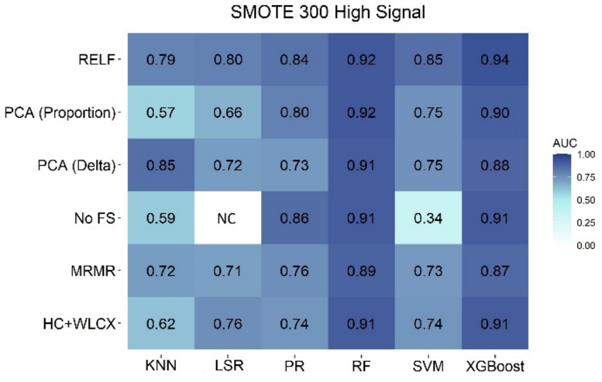

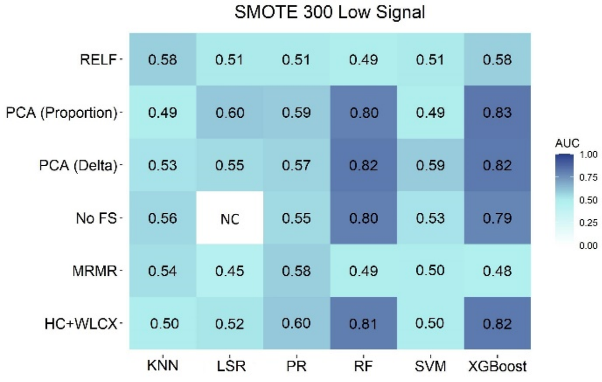

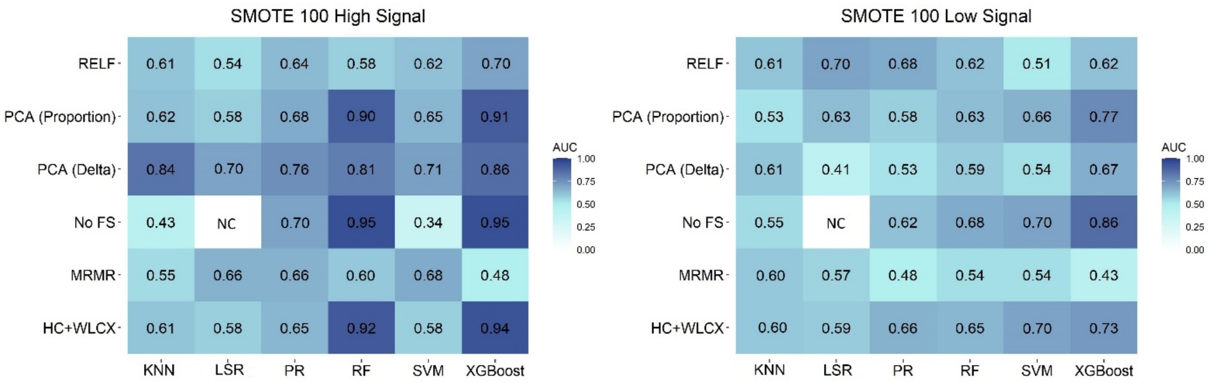

Figure S7. Heatmap representing the predictive performance (AUC) in the validation set for unbalanced SMOTE samples. Performances for feature selection (rows) and classification (columns) methods are displayed after applying SMOTE correction with high signal (panel A) and low signal (panel B).

Table S1. Baseline characteristics of the original study population [1].

Characteristic	All patients (*N* = 270)<br>*N* (%)
**Age (years)^**	67.4 (61.0–72.6)
**Gender**	
Female	103 (38%)
Male	167 (62%)
**Grading**	
1	30 (13%)
2	82 (36%)
3	117 (51%)
Missing	41
**Side**	
Right	153 (57%)
Left	117 (43%)
**Site**	
Upper	154 (57%)
Medium	12 (4%)
Lower	93 (34%)
Mixed	11 (4%)
**Nodule size (mm)^**	31 (18-45)
**pT**	
0	3 (1%)
1	97 (36%)
2	124 (46%)
3	46 (17%)
**pN**	
pN0	199 (74%)
pN1	71 (26%)
**Algorithm type**	
FBP	187 (69%)
IR	83 (31%)

FBP=Filtered Back Projection; IR=Iterative Reconstructions.

^ Median (InterQuantile Range).

Table S2. Wilcoxon p-values for the association of features F1, F2 and F3 with outcome. The test was performed separately for FBP and IR features on the high and low signal scenarios. The translation factors k that was applied to generate the signal are also reported.

Feature	High signal			Low signal		
	k	FBP	IR	k	FBP	IR
F1	1/4	9.55×10^{-26}	5.79×10^{-7}	1/10	9.29×10^{-15}	1.70×10^{-2}

F2	1/100	1.47×10^{-7}	2.04×10^{-5}	1/500	1.00×10^{-1}	2.76×10^{-1}
F3	1/10	4.33×10^{-39}	1.48×10^{-2}	1/50	7.17×10^{-5}	5.83×10^{-1}

References

1. Botta, F.; Raimondi, S.; Rinaldi, L.; Bellerba, F.; Corso, F.; Bagnardi, V.; Origgi, D.; Minelli, R.; Pitoni, G.; Petrella, F.; et al. Association of a CT-based clinical and radiomics score of non-small cell lung cancer (NSCLC) with lymph node status and overall survival. *Cancers (Basel).* **2020**, *12*, 1–16, doi:10.3390/cancers12061432.
2. Vale, C.D.; Maurelli, V.A. Simulating multivariate nonnormal distributions. *Psychometrika* **1983**, *48*, 465–471, doi:10.1007/BF02293687.
3. Vigneau, E.; Chen, M.; Qannari, E.M. ClustVarLV: An R package for the clustering of variables around latent variables. *R J.* **2015**, *7*, 134–148, doi:10.32614/rj-2015-026.
4. Giannitto, C.; Marvaso, G.; Botta, F.; Raimondi, S.; Alterio, D.; Ciardo, D.; Volpe, S.; Piano, F. De Ancona, E.; Tagliabue, M.; et al. Association of quantitative MRI-based radiomic features with prognostic factors and recurrence rate in oropharyngeal squamous cell carcinoma. *Neoplasma* **2021**, *67*, 1437–1446, doi:10.4149/neo_2020_200310N249.
5. Gugliandolo, S.G.; Pepa, M.; Isaksson, L.J.; Marvaso, G.; Raimondi, S.; Botta, F.; Gandini, S.; Ciardo, D.; Volpe, S.; Riva, G.; et al. MRI-based radiomics signature for localized prostate cancer: a new clinical tool for cancer aggressiveness prediction? Sub-study of prospective phase II trial on ultra-hypofractionated radiotherapy (AIRC IG-13218). *Eur. Radiol.* **2021**, *31*, 716–728, doi:10.1007/s00330-020-07105-z.
6. Kuhn, M. Building predictive models in R using the caret package. *J. Stat. Softw.* **2008**, *28*, 1–26, doi:10.18637/jss.v028.i05.
7. Chawla, N. V.; Bowyer, K.W.; Hall, L.O.; Kegelmeyer, W.P. SMOTE: Synthetic Minority Over-sampling Technique. *J. Artif. Intell. Res.* **2002**, *16*, 321–357, doi:10.1613/jair.953.
